# *trans*-Dichloro(triphenylarsino)(*N*,*N*-dialkylamino)platinum(II) Complexes: In Search of New Scaffolds to Circumvent Cisplatin Resistance

**DOI:** 10.3390/molecules27030644

**Published:** 2022-01-19

**Authors:** Mariafrancesca Hyeraci, Laura Agnarelli, Luca Labella, Fabio Marchetti, Maria Luisa Di Paolo, Simona Samaritani, Lisa Dalla Via

**Affiliations:** 1 Department of Pharmaceutical and Pharmacological Sciences, Università degli Studi di Padova, Via F. Marzolo 5, 35131 Padova, Italy; mariafrancesca.hyeraci@studenti.unipd.it; 2 Department of Chemistry and Industrial Chemistry, University of Pisa, Via G. Moruzzi 13, 56124 Pisa, Italy; laura.agnarelli@cpfs.mpg.de (L.A.); luca.labella@unipi.it (L.L.); fabio.marchetti1950@unipi.it (F.M.); simona.samaritani@unipi.it (S.S.); 3 CISUP—Center for the Integration of Scientific Instruments, University of Pisa, 56126 Pisa, Italy; 4 Department of Molecular Medicine, Università degli Studi di Padova, Via G. Colombo 3, 35131 Padova, Italy; marialuisa.dipaolo@unipd.it

**Keywords:** platinum, organometallic complexes, antiproliferative activity, drug resistance

## Abstract

The high incidence of the resistance phenomenon represents one of the most important limitations to the clinical usefulness of cisplatin as an anticancer drug. Notwithstanding the considerable efforts to solve this problem, the circumvention of cisplatin resistance remains a challenge in the treatment of cancer. In this work, the synthesis and characterization of two *trans*-dichloro(triphenylarsino)(*N*,*N*-dialkylamino)platinum(II) complexes (**1** and **2**) were described. The trypan blue exclusion assay demonstrated an interesting antiproliferative effect for complex **1** in ovarian carcinoma-resistant cells, A2780cis. Quantitative analysis performed by ICP-AES demonstrated a scarce ability to platinate DNA, and a significant intracellular accumulation. The investigation of the mechanism of action highlighted the ability of **1** to inhibit the relaxation of supercoiled plasmid DNA mediated by topoisomerase II and to stabilize the cleavable complex. Cytofluorimetric analyses indicated the activation of the apoptotic pathway and the mitochondrial membrane depolarization. Therefore, topoisomerase II and mitochondria could represent possible intracellular targets. The biological properties of **1** and **2** were compared to those of the related *trans*-dichloro(triphenylphosphino)(*N*,*N*-dialkylamino)platinum(II) complexes in order to draw structure–activity relationships useful to face the resistance phenotype.

## 1. Introduction

The clinical use of cisplatin, the first platinum-based anticancer drug, was approved by the FDA in 1978 for the treatment of ovarian, testicular and bladder cancer. Since then, its effectiveness against different types of cancers, including colorectal and non-small cell lung cancer, neuroblastoma and mesothelioma, was proven [1].

Nevertheless, the appearance of side effects, mainly nephro- [2,3] and oto-toxicity [4,5], and the development of intrinsic or acquired resistance [6,7], markedly reduced the clinical use of cisplatin as anticancer drug. Platinum drug resistance is a complex multifactorial phenomenon, resulting from different mechanisms, such as the reduction of intracellular drug accumulation due to the downregulation of transporters and/or to the upregulation of proteins involved in its detoxification or efflux, the failure of the apoptotic pathways and the increase in repair of DNA damage [6,8,9,10]. Many efforts were devoted to the development of platinum compounds with reduced undesired toxicity, with carboplatin [11] and oxaliplatin [12] reaching the clinic in 1989 and 2002, respectively, demonstrating to be safer than cisplatin [13]. Otherwise, circumventing resistance has proven to be a difficult goal to achieve for anticancer therapy and still represents an intriguing challenge for researchers and clinicians [13,14].

**Scheme 1:**
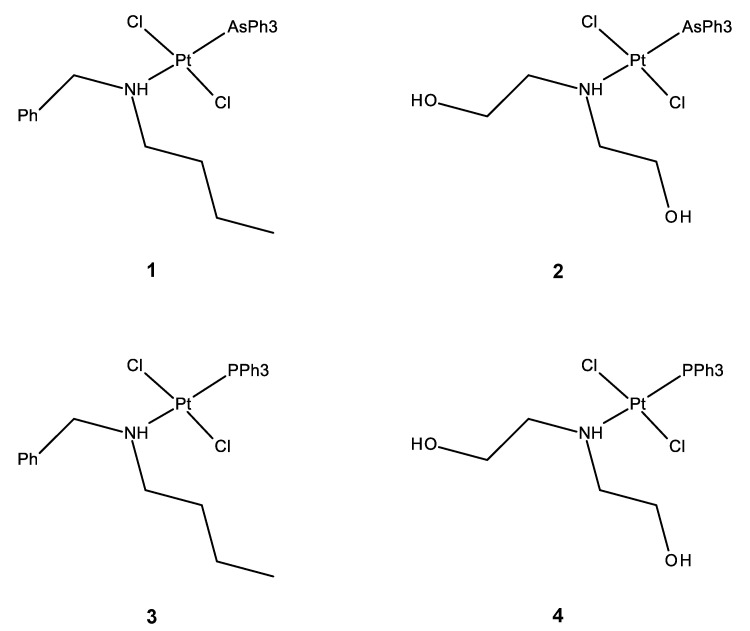
Complexes **1** and **2** and previously studied triphenylphosphine analogues 3 [18] and 4 [22].

Over the past few years, novel Pt(II) complexes sharing a common triphenylphosphine ligand have been synthesized [15,16,17,18,19,20,21,22]. The investigation of the mechanism of action of some of these complexes underlined an interesting biological profile characterized by significant cytotoxicity in the ovarian carcinoma cell line resistant to cisplatin and by the ability to interact with various intracellular targets [18,19,20,21,22]. In particular, we collected homogeneous data concerning the mode of action of complexes 3 [18] and 4 [22] (Scheme 1), which, among the complexes described [18,22], were the most active. Considering that the high-yield synthesis of complexes 3 and 4 could also be easily exploited for the preparation of structural analogues where triphenylphosphine was replaced by triphenylarsine, we chose 3 and 4 as useful models for a comparative study. On this basis and in order to identify possible new scaffolds involved in overcoming resistance, we designed and synthesized new Pt(II) complexes **1** and **2**, characterized by the triphenylarsine ligand, and we chose to maintain in the coordination sphere of the metal, besides two chlorido ligands, the same dialkylamino residues as in 3 and 4, in order to easily compare the effect of replacing phosphorus with arsenic (Scheme 1).

Although triphenylphosphine and triphenylarsine share several characteristics, such as for the coordination mode to metals, it is known that the heavier analogue shows a less strong *trans* effect in platinum(II) complexes [23,24]. This feature could be the basis for a different reactivity towards biological substrates and, hence, for a different cytotoxicity profile. Few studies have reported about metallodrugs containing a triphenylarsine ligand. In most cases, triphenylarsine is a co-ligand, and the investigation is mainly focused on the effect of other residues in coordination with the metal [25,26,27,28,29]. Nevertheless, interesting results are described for some dichloroplatinum(II) complexes bearing two aromatic arsino ligands and hydroxy and/or methoxy substituents on one of the three aromatic moieties. These complexes proved a cytotoxicity profile against ovarian cancer cells comparable to that of carboplatin [30].

To assay the cytotoxicity of the tested complexes, in addition to the pair of cisplatin-sensitive and -resistant cell lines (A2780 and A2780cis, respectively), this study also considered three other human tumor cell lines, HeLa, HT-29 and A549. The covalent interaction with DNA and the effect on the nuclear topoisomerase IIα enzyme were investigated to identify intracellular targets responsible for the cytotoxicity. The uptake, the effect on mitochondria and the apoptotic pathway were evaluated in ovarian carcinoma-resistant and -sensitive cells. The results are discussed in comparison with the biological properties of the triphenylphosphine analogues.

## 2. Results and Discussion

### 2.1. Synthesis

**Figure 1:**
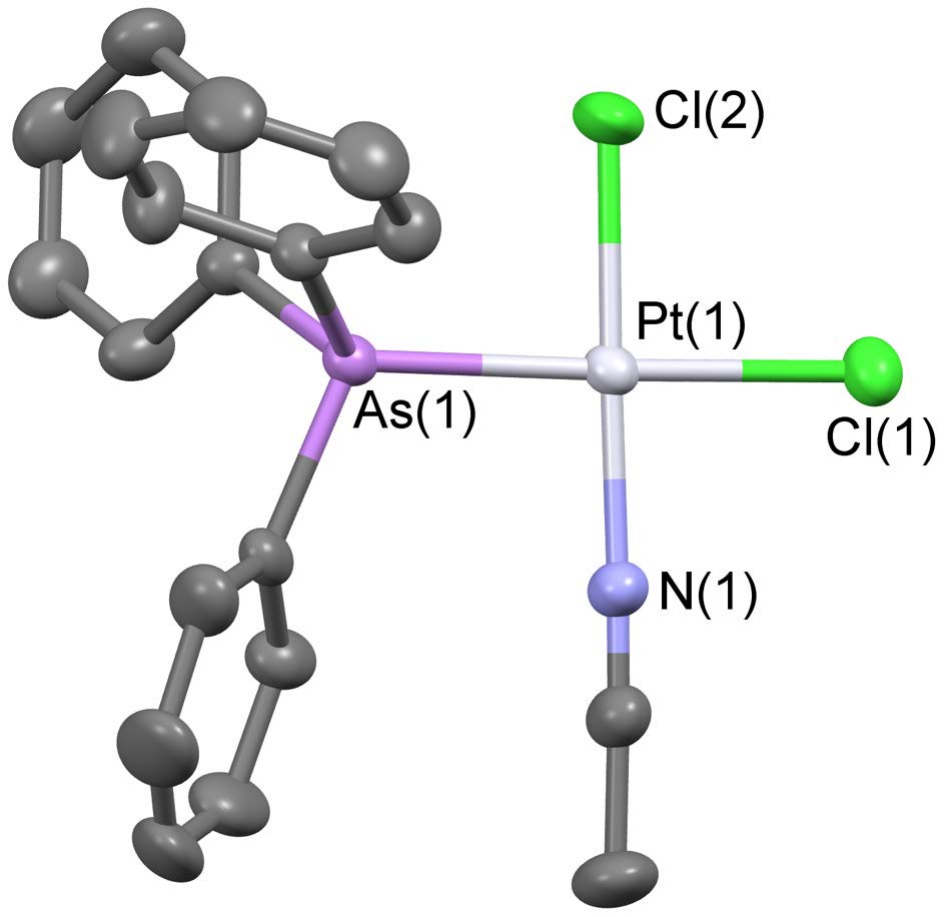
View of the molecular structure of *cis*-[PtCl_2_(AsPh_3_)(NCMe)]. Thermal ellipsoids are at 30% probability.

The synthesis of *trans*-dichloro(*N*-butyl,*N*-benzylamino)(triphenylarsino)platinum(II) (**1**) and *trans*-dichloro(*N*,*N*-*bis*(2-hydroxyethyl)amino)(triphenylarsino)platinum(II) (**2**) was carried out by bridge-splitting of *trans*-[Pt(μ-Cl)Cl(AsPh_3_)]_2_ by the suitable amines. The di-nuclear precursor was prepared from [PtCl_2_(NCMe)_2_] and triphenylarsine in acetonitrile under solvothermal conditions (130 °C). The reaction afforded crystalline *cis*-[PtCl_2_(AsPh_3_)(NCMe)] (Figure 1, for experimental details see Materials and Methods, Table 3). Although the procedure was previously optimized for the preparation of cis-[PtCl_2_(PPh_3_)(NCEt)] [31], it is worth noting that the preparation of the arsenic derivative requested a lower temperature (130 vs. 155 °C) and was faster (3 vs. 120 h) than the reaction affording the phosphorus counterpart.

In Table 1, selected bond lengths and angles are reported for *cis*-[PtCl_2_(AsPh_3_)(NCMe)].

The coordination around platinum is square planar, with a *cis* configuration and small deviations from ideality. Crystal data for this complex can be compared with the analogous parameter reported for *cis*-[PtCl_2_(PPh_3_)(NCEt)] [31]. In particular, there is a good agreement between crystal data in the two complexes for Pt-N and Pt-Cl bond lengths, while, as expected, the Pt-As bond length is significantly longer than that for Pt-P (2.3610(4) vs. 2.2355(19), respectively). The complex was than refluxed in toluene, where it promptly released coordinated acetonitrile to form the di-nuclear *trans*-[Pt(μ-Cl)Cl(AsPh_3_)]_2_ in a nearly quantitative yield. Complexes **1** and **2** (Scheme 1) were obtained by a well-known ring-opening reaction of di-nuclear *trans*-[Pt(μ-Cl)Cl(AsPh_3_)]_2_ precursor by the suitable amines, guided by the *trans* effect of the Ph_3_As ligand, affording *trans* products. The complexes were recovered with very good, isolated yields (81% and 83%, respectively). Both **1** and **2** are insoluble in water and well-soluble in DMSO, where partial isomerization from a *trans* to *cis* isomer was observed by ^1^H NMR analysis (Supplementary Materials Figure S9) in 24 h.

### 2.2. Antiproliferative Activity

The cytotoxicity of **1** and **2** was assessed using a trypan blue dye exclusion assay. Cell lines, HeLa (cervix adenocarcinoma), HT-29 (colon adenocarcinoma), A549 (lung carcinoma), A2780 (ovarian carcinoma), A2780cis (ovarian carcinoma resistant to cisplatin) and Met-5A (mesothelial cells), were incubated for 72 h in the presence of increasing concentrations of the tested complexes, and the GI_50_ values (concentration of complex inducing a 50% reduction in cell growth) were calculated and shown in Table 2.

Cisplatin was tested as a reference drug and 3 and 4, the analogues containing the phosphorus counterpart, were used for comparison.

After 72 h of incubation, complexes **1** and **2** induced a significant cytotoxicity, leading to GI_50_ values ranging mainly in the low micromolar range.

Complex **1** appears to be more effective than **2** in all tested cell lines, underlining a relevant role for the *N*,*N*-dialkylamino side chains and suggesting a favorable contribution for the more hydrophobic residues inserted in **1**. Interestingly, complex **1** shows a lower cytotoxic effect on non-tumorigenic mesothelial cells, Met-5A, with respect to ovarian carcinoma cells, A2780. Furthermore, the comparison with the antiproliferative activity observed for the corresponding triphenylphosphine complex 3 [18] highlighted the triphenylarsine ligand as more effective in inducing the cytotoxic effect on ovarian carcinoma. In fact, the GI_50_ values obtained for the newly synthesized **1** are seven and two times lower than those of 3, in sensitive and resistant ovarian carcinoma cells, respectively. Finally, the cytotoxic effect exerted by **1** in ovarian carcinoma cells is higher with respect to that exerted by cisplatin, with GI_50_ values two- (A2780) and three-fold (A2780cis) lower than that of the reference drug.

Complex **2** was markedly less effective than **1** in inducing cytotoxicity. Moreover, the substitution of the triphenylphosphine ligand (complex 4) with the triphenylarsine (complex **2**) seems to be detrimental to the biological effect since GI_50_ values were about twenty and seven times higher than those of the analogue 4 in HeLa and A549 cells, respectively [22].

### 2.3. Interaction with DNA

**Figure 2:**
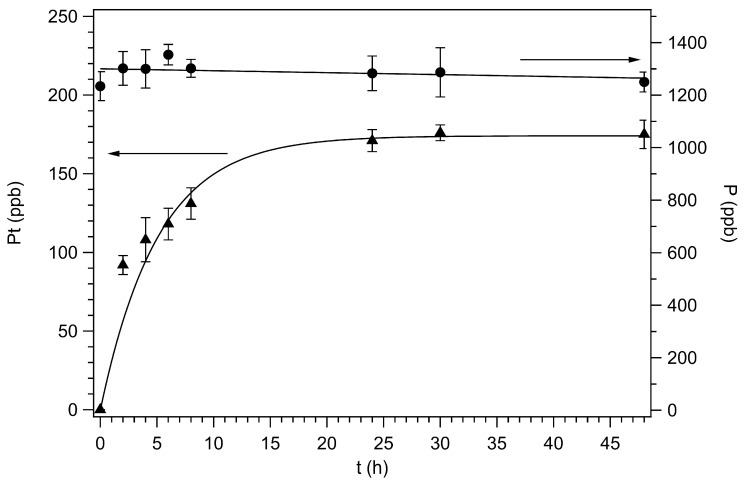
Quantitative analysis of platinum (triangles) and phosphorus (circles) bound to DNA performed by ICP-AES. Salmon testes DNA (9 × 10^−4^ M) was incubated with **1** for different durations (0–48 h) at [DNA]/[complex] = 10. Mean values ± SD of three experiments in duplicate are reported.

The results in Table 2 and in particular the cytotoxic effect exerted on A2780 and A2780cis cells prompted us to further investigate the biological properties of complex **1**. As the ability to form DNA-platinum adducts accounts for the anticancer effect and the clinical effectiveness of cisplatin, we evaluated the capacity of **1** to covalently bind to salmon testes DNA (Figure 2).

In detail, the macromolecule was incubated in the presence of **1**, and at different fixed incubation times (0–48 h), aliquots of solution were collected and analyzed by ICP-AES to determine the platinum content.

The phosphorus amount was also analyzed and taken as an internal standard for each sample, as previously reported [18]. The obtained results, expressed as ppb (Figure 2), indicate the capability of the new complex **1** to covalently add to DNA, with a saturating behavior that reached the plateau after 24 h. The maximum amount of Pt detected was about 180 ppb (4.6 nmol), a value comparable to that obtained for the corresponding triphenylphosphine complex 3 (about 200 ppb), and significantly lower than that of the reference drug, cisplatin (about 800 ppb) [18]. These results suggest that the reactivity toward the macromolecule is similarly affected by the two ligands, triphenylphosphine and triphenylarsine. Moreover, both **1** and 3 bound to the macromolecule in a significantly lower amount with respect to the reference drug.

### 2.4. Effect on Topoisomerase II Catalytic Cycle

**Figure 3:**
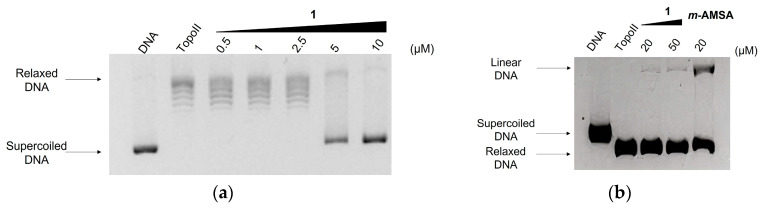
Effect of complex **1** on (**a**) relaxation of supercoiled DNA by topoisomerase II and (**b**) stabilization of covalent DNA-topoisomerase II complex. Supercoiled plasmid pBR322 (DNA) was incubated with topoisomerase II in the absence (Topo II) and in the presence of complex **1** at indicated concentrations. 20 μM *m*-AMSA was used as a poison reference drug.

Based on these considerations, the cytotoxicity induced by 3 in resistant A2780cis cells has been related to the ability of the complex to interfere with the catalytic activity of topoisomerase II [18], a nuclear enzyme able to solve the topological problems that arise in DNA metabolism. In fact, it was reported that replication, transcription and sister chromatid segregation require the involvement of this enzyme [32]. Moreover, several studies explored a possible role of topoisomerase II in human cancers, and for some malignancies, elevated expression of the enzyme has been associated with poor prognosis [33,34,35,36]. In particular, in ovarian carcinoma, a correlation between high levels of topoisomerase II and short overall survival was revealed, and in addition, an unfavorable prognostic role was documented in the subgroup of platinum-resistant ovarian cancer patients [37]. More recently, an upregulation of topoisomerase II was demonstrated in high-grade serous ovarian cancer, the most aggressive form of ovarian cancer, and the enzyme expression appears to be related to proliferation, migration and invasion capacities [38]. In this connection, it appeared of interest to investigate the effect of complex **1** on the relaxation of supercoiled plasmid DNA catalyzed by topoisomerase II, and the results are shown in Figure 3a. In the presence of the enzyme (lane Topo II), supercoiled pBR322 DNA (lane DNA) is fully converted into the relaxed forms. In the presence of complex **1** at a 5 μM concentration, the relaxation activity mediated by topoisomerase II appears to be significantly inhibited, as demonstrated by the decrease of the bands corresponding to the relaxed forms and the appearance of the native supercoiled DNA. The disappearance of the relaxed forms observed at 10 μM demonstrates the complete inhibition of the enzyme. By comparing the above results with those obtained for 3, for which no appreciable effect was previously observed at the 5 μM concentration [18], it is possible to ascribe a more pronounced inhibitory activity to **1** than the corresponding triphenylphosphine analogue.

The agents that affect the catalytic cycle of topoisomerase II fall into two main groups: catalytic inhibitors and poisons. The catalytic inhibitors are compounds that interfere with different steps of the catalytic cycle, lowering the activity of the enzyme. The poisons render the enzyme unable or less efficient in relegation of DNA-strand breaks, stabilizing a covalent intermediate (cleavable complex) and leading to the accumulation of enzyme-mediated double-strand breaks [39]. The occurrence of such damage was experimentally demonstrated by the enzyme-mediated formation of linear DNA from supercoiled DNA. Figure 3b shows the results obtained by incubating supercoiled pBR322 DNA with the enzyme in the presence of complex **1** at 20 and 50 μM concentrations and performing the electrophoretic run in agarose gel containing ethidium bromide. The well-known topoisomerase II poison *meta*-amsacrine (*m*-AMSA) was used as a reference at a 20 μM concentration. Interestingly, **1** appeared able to behave as topoisomerase II poison, as demonstrated by the occurrence of linear DNA (Figure 3b). Taking into account that double-strand breaks are lethal damage for the cells and considering the crucial role played by topoisomerase II in ovarian carcinoma proliferation and aggressiveness, it is possible to hypothesize that the poisoning effect exerted by **1** on the enzyme could contribute to the cytotoxic mechanism observed in A2780 and A2780cis cells (Table 2).

### 2.5. Uptake in Ovarian Carcinoma Cells

**Figure 4:**
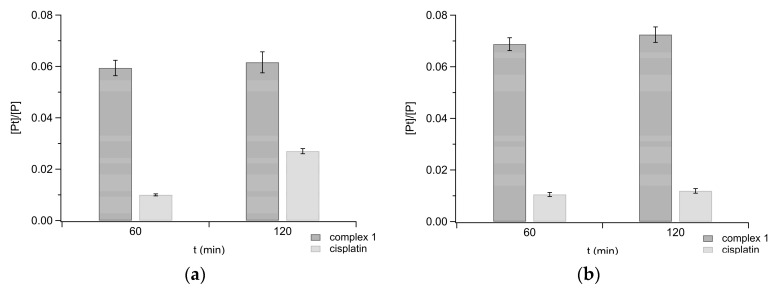
Platinum (ppb)/Phosphorus (ppb) ratio ([Pt]/[P]) in A2780 (**a**) and A2780cis (**b**) cells incubated with 100 μM **1** for 60 and 120 min. Cisplatin is shown as a reference. Mean values ± SD of at least three experiments in duplicate are reported.

The significant antiproliferative effect exerted by **1** on resistant A2780cis cells suggests that the complex may be able to overcome some mechanisms involved in the resistance phenomenon, such as low cell accumulation [13,40]. In this connection, we evaluated the cell uptake of **1** by ICP-AES by measuring the amount of Pt (ppb) in A2780 and A2780cis cells incubated for 60 and 120 min with the test complex. In the same samples, the amount of P (ppb) was also analyzed and taken as an internal standard, because it is related to the cell number, and the obtained data were expressed as [Pt]/[P] (Figure 4).

In cells incubated with **1**, a remarkable Pt content was found, notably higher with respect to that of the reference drug, suggesting for the new complex the ability to easily cross the plasma cell membrane. It can be hypothesized that such capacity comes from the high lipophilicity of the new complex, a property that could also explain the significant difference with respect to the uptake of cisplatin in both cell lines [20,21,41]. It is worth noting that for cisplatin accumulation in cancer cells, multiple carrier proteins have been proposed [13,42]. Otherwise, based on the above reported results, an uptake mediated by passive diffusion could be suggested for **1**. Therefore, resistance mechanisms related to loss or impairment of the transporter mechanism(s) associated with cisplatin accumulation could not be involved in the uptake of **1**.

### 2.6. Mitochondria Damage and Cell Death

**Figure 5:**
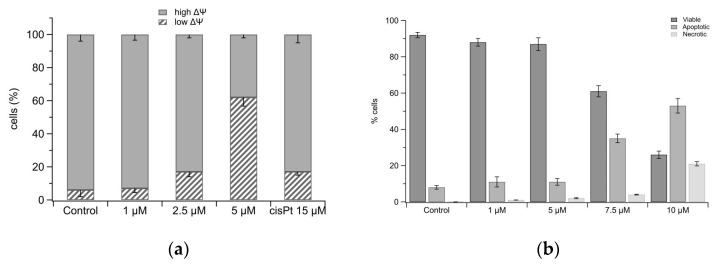
Cytofluorimetric analyses in A2780cis cells (3 × 10^5^) incubated for 40 h with complex **1** or cisplatin (cisPt) at indicated concentrations. (**a**) Mitochondrial transmembrane potential (ΔΨ) in cells loaded with JC-1 (5,5′,6,6′-tetrachloro-1,1′,3,3′-tetraethylbenzimidazolcarbocyanine iodide). (**b**) Percentage of viable, apoptotic and necrotic cells loaded with Annexin V-FITC and propidium iodide. Values are the mean ± SD of four independent experiments.

The antiproliferative activity exerted by some *N*,*N*-dialkylaminotriphenylphosphine Pt(II) complexes has been previously related to the ability to impair mitochondria by damaging mitochondrial membrane permeability [21,22]. In this connection, the effect of **1** on mitochondria was also investigated. For this purpose, ovarian carcinoma-resistant A2780cis cells were incubated for 40 h with **1** and then stained with the membrane-permeant cationic probe JC-1, to be analyzed by flow cytometry (Figure 5a and Supplementary Materials Figure S10). The performed assay allows to evaluate mitochondrial transmembrane potential (ΔΨ) and to distinguish mitochondria in whole cells with high ΔΨ (healthy) from mitochondria with low or collapsed ΔΨ (damaged). As shown in Figure 5a, at a 5 μM concentration, **1** induces the collapse of ΔΨ in about 60% of cells. In the presence of cisplatin, such damage occurs to a significantly lesser extent, and indeed at 15 μM, the reference drug induces mitochondria depolarization in about 20% of cells. These results suggest a relationship for **1** between mitochondria damage and cytotoxicity in resistant cells, suggesting the organelles as a possible intracellular target. The ability of **1** to affect mitochondria functionality was also confirmed in A2780 cells (Supplementary Materials Figure S11).

Mitochondria exert a pivotal role both in vital and lethal cell functions. The organelles indeed represent the powerhouse of eukaryotic cells, by producing ATP through the oxidative phosphorylation, and also participate in the apoptotic signaling by releasing, following cell damage, pro-apoptotic factors in the cytosol [43]. The ability of complex **1** to provoke transmembrane depolarization suggests the involvement of the damaged organelles in promoting cell death through the apoptotic pathway. Therefore, the occurrence of apoptosis following cell treatment with **1** has been investigated by a cytofluorimetric assay (Figure 5b and Supplementary Materials Figure S12). In detail, A2780cis cells were incubated for 40 h with test complex and stained with Annexin V-FITC and propidium iodide. Figure 5b shows the percentage of viable cells (Annexin V-negative/PI-negative) and of cells undergoing apoptosis (Annexin V-positive/PI-negative and Annexin V-positive/PI-positive) or necrosis (Annexin V-negative/PI-positive). Complex **1** appeared to be able to induce a dose-dependent increase in apoptotic cells, which reached a percentage of about 50% at 10 μM. It is interesting to note that at 5 μM, the tested complex promoted more than 60% of mitochondria depolarization, and induced apoptosis in less than 20% of cells.

The apoptotic effect of complex **1** is more evident on the sensitive A2780 cells, and indeed, at a 10 μM concentration, about 80% of cells undergo apoptosis (Supplementary Materials Figure S13), in agreement with the GI_50_ values (Table 2).

Based on these results, it is possible to hypothesize the mitochondrial membrane depolarization as the starting point for the apoptosis signaling in resistant cells, thus supporting the proposal of mitochondria as a possible cell target for complex **1**.

## 3. Materials and Methods

### 3.1. General Information

Synthetic procedures were performed under a dinitrogen atmosphere. All solvents used were dried over molecular sieves and stored under a dinitrogen atmosphere [44]. CDCl_3_ solutions, if not otherwise stated, were used to register ^1^H-, ^13^C{H}- and ^195^Pt{H}-NMR spectra with a Bruker “Avance DRX400” spectrometer. TMS was used to reference ^1^H and ^13^C chemical shifts (δ ppm), while aqueous (D_2_O) H_2_PtCl_6_ was used for ^195^Pt. When samples were dissolved in non-deuterated solvents, the instrumental lock to the deuterium signal was achieved by inserting a sealed capillary containing C_6_D_6_ into the NMR tube. FTIR spectra in attenuated total reflectance (ATR) were registered with a Perkin–Elmer “Spectrum One” spectrometer. Band intensities are described as follows: w (weak), m (medium) and s (strong). Elemental analyses (C, H, N) were performed using an Elementar “vario MICRO cube” instrument, at Dipartimento di Chimica e Chimica Industriale, Università di Pisa.

Benzylbutylamine (BzBuNH) was synthesized from benzaldehyde and butylamine [18] and distilled before use. Diethanolamine (™Fluka) was passed over dry alumina before use.

### 3.2. Preparation and Characterization of New Complexes

#### 3.2.1. Synthesis of *cis*-[PtCl_2_(AsPh_3_)(NCMe)]

A mixture of [PtCl_2_(NCMe)_2_] (0.4000 g, 1.147 mmol), AsPh_3_ (0.3520 g, 1.149 mmol) and acetonitrile (10 mL) was heated (130 °C) in a 100 mL Carius tube until a clear yellow solution was obtained (3 h). The solution was slowly cooled at room temperature (25 °C). Pale yellow crystals formed were recovered by filtration and dried under vacuum (0.299 g, 42.5% yield). Single-crystal X-ray diffraction was carried out on a selected crystal and showed the *cis* configuration of the complex. ^1^H NMR: 7.19–7.72 (m, 15 H), 1.78 (s, 3H, NCCH_3_, ^4^J_H-Pt_ = 15 Hz). ^195^Pt-NMR: –3395 (*cis* isomer). C_20_H_18_AsCl_2_Pt requires: C 39.17, H 2.96, N 2.28. Experimental: C 39.35, H 3.07, N 2.15%. A solid residue was obtained eliminating the solvent from mother liquors of the preparation (0.145 g), containing a mixture of *cis,trans*-[PtCl_2_(AsPh_3_)(NCMe)]. Selected signals from the mixture spectra for the *trans* complex showed: ^1^H NMR: 7.18–7.77 (m, 15H, H arom), 2.35 (s, 3 H, NCCH_3_); ^195^Pt NMR: −3331. Crystal data can be found in the table below (Table 3).

#### 3.2.2. Synthesis of *trans*-[Pt(µ-Cl)Cl(AsPh_3_)]_2_

A mixture of [PtCl_2_(NCMe)_2_] (1.152 g, 3.30 mmol), AsPh_3_ (1.012 g, 3.30 mmol) and acetonitrile (15 mL) was heated (130 °C) in a 100 mL Carius tube. A clear, yellow solution was obtained, and heating was continued for 3 h. The solution was then concentrated to dryness under vacuum and the yellow residue was suspended in toluene (30 mL). The suspension was refluxed (110 °C) until the complete precipitation of a brilliant orange solid (3.598 g, 95% yield) was observed (2 h). The product (4.72 g, 80% yield) was recovered by filtration and dried under vacuum. C_36_H_30_As_2_Cl_4_Pt_2_ requires: C 37.78, H 2.64%. Experimental: C 37.69, H 2.83%. IR (ATR) cm ^1^: 3053 m, 1964 s, 1579 m, 1481 s, 1434 s, 1309 m, 1189 m, 1160 m, 1080 s, 1022 m, 998 s, 740 s, 690 s.

#### 3.2.3. Synthesis of *trans*-[PtCl_2_(AsPh_3_)(NHBzBu)] (**1**)

A sample of *trans*-[Pt(µ-Cl)Cl(AsPh_3_)]_2_ (0.350 g, 0.306 mmol) was suspended in 1,2-dichloroethane (10 mL) and treated under stirring with 0.518 g of benzylbutylamine (0.705 mmol, amine/Pt = 1.15 molar ratio). A yellow solution was obtained almost immediately. After 1 h, the solution was concentrated under vacuum up to one fourth of the original volume and heptane was added (20 mL). The yellow solid obtained was then recovered by filtration and dried under vacuum (0.364 g, 81% yield). C_29_H_32_Cl_2_NAsPt·H_2_O requires: C 46.23, H 4.55, N 1.86%. Experimental: C 46.42, H 4.34, N 1.87%. ^1^H NMR: 7.68–7.37 (m, 20H, AsPh_3_ + CH_2_Ph); 4.63 (dd, 1H, J = 12.9, J′ = 6.4 Hz, NHCHHPh), 4.25 (bm, 1H, ^2^J_H-Pt_ = 72 Hz, NH), 3.93 (dd, J = 12.9, J′ = 7.8 Hz, NHCHHPh), 3.34 (m, 1H, NHCHHCH_2_), 2.63 (m, 1H, NHCHHCH_2_), 2.36 (m, 1H, NHCH_2_CHH), 1.94 (m, 1H, NHCH_2_CHH), 1.49 (m, 1H, NHCH_2_CH_2_CHH), 1.40 (m, 1H, NHCH_2_CH_2_CHH), 0.97 (t, J = 7.4 Hz, CH_3_). ^13^C NMR: 135.8, 134.0, 130.4, 130.1, 130.0, 128.8, 128.5, 128.3, 57.0, 51.1, 31.1, 20.0, 13.9. ^195^Pt NMR: −3347.

#### 3.2.4. Synthesis of *trans*-[PtCl_2_(AsPh_3_)(NH(CH_2_OH)_2_)] (**2**)

A sample of *trans*-[Pt(µ-Cl)Cl(AsPh_3_)]_2_ (0.290 g, 0.254 mmol) was suspended in 1,2-dichloroethane (10 mL) and treated under stirring with 55.9 μL of diethanolamine (0.507 mmol, amine/Pt = 1.15 molar ratio). A pale-yellow solution was obtained almost immediately. After 1 h, the solvent was eliminated under vacuum and the oily residue was treated with petroleum ether (20 mL) overnight, under stirring at room temperature. The yellow solid obtained was recovered by filtration and dried under vacuum (0.283 g, 83% yield). C_22_H_26_Cl_2_NO_2_AsPt·H_2_O requires: C 38.00, H 4.06, N 2.01%. Experimental: C 38.03, H 3.91, N 2.18%. ^1^H NMR: 7.73–7.31 (m, 15H, AsPh_3_); 4.97 (m, 3H, OCH_2_ + NH), 3.92 (m, 2H, OCH′_2_), 3.43 (m, 2H, NHCH_2_), 2.83 (m + bs, 4H, NHCH′_2_ + OH). ^13^C NMR: 133.8, 130.7, 129.6, 128.7, 60.4, 55.1. ^195^Pt NMR: −3349.

### 3.3. Cell Cultures

A2780 (human ovarian carcinoma) and A2780cis (cisplatin-resistant human ovarian carcinoma) were purchased from the European Collection of Cell Cultures, UK (ECACC). HT29 (human colorectal cancer), A549 (human non-small cell lung cancer), HeLa (human cervix adenocarcinoma) and Met-5A (human mesothelium) were purchased from American Type Culture Collection, USA (ATCC). A2780, A2780cis and HT-29 were cultured in RPMI 1640 (Sigma-Aldrich R6504, Merck Life Science S.r.l., Milano, Italy), Met-5A in RPMI 1640 (Sigma-Aldrich R6504, Merck Life Science S.r.l., Milano, Italy) supplemented with 2.38 g/L HEPES, 0.11 g/L pyruvate sodium and 2.5 g/L glucose, A549 in Nutrient Mixture F-12K (Sigma-Aldrich N3520, Merck Life Science S.r.l., Milano, Italy) and HeLa in Nutrient Mixture F-12 Ham (Sigma-Aldrich N6760, Merck Life Science S.r.l., Milano, Italy). All cell media were supplemented with 1.5 g/L NaHCO_3_, 10% heat-inactivated fetal bovine serum (Gibco, Thermo Fisher, Milano, Italy), 100 U/mL penicillin, 100 μg/mL streptomycin and 0.25 μg/mL amphotericin B (Sigma-Aldrich A5955, Merck Life Science S.r.l., Milano, Italy). The cell lines were cultured as monolayers in a humidified atmosphere at 37 °C and 5% CO_2_ and sub-cultured at 80% confluency. The resistant cell line was maintained in medium with 1 μM of cisplatin (Sigma-Aldrich P4394, Merck Life Science S.r.l., Milano, Italy) every 2–3 passages.

### 3.4. Inhibition Growth Assay

The cytotoxicity of the tested complexes was evaluated using the Trypan blue exclusion assay. Briefly, cells were seeded in 24-well plates at the appropriate density (2.5–3 × 10^4^) in each well and incubated with complete growth medium overnight. Then, cells were exposed to different complex concentrations and incubated for 72 h. Complexes **1** and **2** were dissolved in dimethylsulfoxide at 20 mM and diluted in medium so that the final volume of solvent was below 0.5% (*v*/*v*). Cisplatin was dissolved in 0.9% saline at a 4 mM final concentration. Following incubation, cells were washed with phosphate buffered saline (PBS, 0.1 M NaCl, 2 mM KCl, 8 mM Na_2_HPO_4_·2H_2_O, 1.5 mM KH_2_PO_4_), harvested, diluted in an appropriate volume of Trypan blue (0.1%) and counted by an optical microscope. The results were analyzed and the GI_50_ values (micromolar concentration which induces a 50% reduction in cell number with respect to the control condition) were estimated.

### 3.5. Nucleic Acids

The concentration of salmon testes DNA (Sigma-Aldrich, D1626, Merck Life Science S.r.l., Milano, Italy) was determined spectrophotometrically using the molar absorption coefficient ε = 6600 M^−1^ cm^−1^ at 260 nm to express the polynucleotide concentration in phosphate units [45]. pBR322 (SD0041 Thermo Scientific, Milano, Italy) was used as plasmid supercoiled DNA.

### 3.6. Binding to DNA

Measurement of DNA platination was performed according to a previous method [46]. Appropriate volumes of solution of test complex were added to aqueous solution of salmon testes DNA (9 × 10^−4^ M) to achieve a DNA/complex molar ratio of 10. Exact volumes (250 μL) of such solutions were collected at fixed times (0–48 h), and the DNA was precipitated twice with 0.3 M sodium acetate and ice-cold ethanol and washed with 70% ethanol. The pellets were dissolved in milliQ^®^ water, mineralized with concentrated HNO_3_ (65% *v*/*v*) for 1 h at 90 °C in a digital dry bath (Gilson), diluted in 3 volumes of 37% (*v*/*v*) HCl and added with milliQ^®^ water up to 5 mL. The content of P and Pt was analyzed by a Spectroflame Modula sequential and simultaneous ICP-spectrometer (ICP SPECTRO Arcos with EndOnPlasma torch) equipped with a capillary cross-flow Meinhard nebulizer (Spectro Analytical Instruments, Kleve, Germany). Emission lines λ = 178.290 nm and λ = 214.423 nm were used to determine P and Pt, respectively. A sample undergoing the same experimental procedure and containing DNA alone was also analyzed, and confirmed the absence of Pt as a possible contaminant. Calibration was carried out by preparing 5 multi-element standard solutions containing Pt and P in the concentration range 0–2 mg L^−1^ for Pt and 0–10 mg L^−1^ for P (ppm). Standard solutions were prepared by diluting Pt (standards from Romnil) and P (Spectrascan standards from Teknolab) stock solutions of 1000 mg L^−1^ with HCl 2.5% *v/v* and HNO_3_ 2.5% *v*/*v*, respectively.

### 3.7. DNA Topoisomerase Relaxation Assay

The topoisomerase II relaxation activity assay was performed in a 20 μL total volume of reaction buffer containing 5 mM TrisHCl (pH 7.5), 12.5 mM NaCl, 1 mM MgCl_2_, 5 mM DTT, 10 μg/mL albumin, 1 mM ATP, 1 U Topoisomerase IIα (Inspiralis, Norwich, UK) and 250 ng supercoiled plasmid DNA. The reactions were performed for 1 h at 37 °C in the presence of the investigated complex at indicated concentrations. Finally, reactions were stopped by adding 4 µL of stop buffer containing 5% SDS, 0.125% bromophenol blue, 25% glycerol and 50 µg/mL proteinase K (Sigma-Aldrich) and incubating for a further 30 min at 37 °C. The samples (15 μL) were then subjected to electrophoresis on 1% agarose gel in TAE (0.04 M TRIS, 0.02 M glacial acetic acid and 1 mM EDTA, pH 8) at 100 V for 90 min. The gels were stained in TAE buffer containing 1 μg/mL of ethidium bromide (Sigma E1510) and photographed using a gel-imaging system equipped with a CCD camera coupled to a Bio-Rad Gel Doc XR apparatus (BioRad, Hercules, CA, USA).

### 3.8. Topoisomerase II-Mediated DNA Cleavage

Supercoiled pBR322 plasmid DNA (250 ng) was incubated with 10 U Topo IIα (Inspiralis, Norwich, UK) for 1 h at 37 °C in 20 µL of reaction buffer (5 mM TrisHCl (pH 7.5), 12.5 mM NaCl, 1 mM MgCl_2_, 5 mM DTT, 10 μg/mL albumin, 1 mM ATP), in the presence of the investigated complex at indicated concentrations. Reactions were terminated by adding 4 µL of stop buffer (5% SDS, 0.125% bromophenol blue and 25% glycerol), 50 µg/mL of proteinase K (Sigma-Aldrich, Merck Life Science S.r.l., Milano, Italy) and by incubating the samples for 30 min at 37 °C. Then, 15 μL of each sample were electrophoresed at 30 V for 18 h in a 1% agarose gel prepared in TBE buffer (0.09 M TRIS-borate and 0.002 M EDTA) containing 0.5 μg/mL of ethidium bromide (Sigma E1510). Gels were de-stained in milliQ^®^ water and photographed under UV light by a CCD camera coupled to a Bio-Rad Gel Doc XR apparatus (BioRad, Hercules, CA, USA).

### 3.9. Cell Uptake

To measure intracellular platinum accumulation, 2 × 10^6^ A2780cis cells were seeded into 6-well plates and allowed to grow in standard conditions for 24 h. Then, the cells were incubated with 100 μM of test complex for 60 and 120 min, washed twice with 0.9% NaCl to remove dead cells and trypsinized. The pellet was washed twice with 0.9% NaCl, mineralized with concentrated HNO_3_ (65%) for 1 h at 90 °C in a digital dry bath (Gilson Srl, Milano, Italy), diluted in 3 volumes of 37% (*v*/*v*) HCl and added with milliQ^®^ water up to 5 mL. The content of P and Pt was analyzed and determined as described in Section 3.6.

### 3.10. Mitochondrial Transmembrane Potential Measurement

The mitochondrial transmembrane potential was evaluated according to Cossarizza et al. [47] and measured by the BD™ MitoScreen Kit (BD Pharmigen). Briefly, resistant A2780cis cells were seeded at a density of 3 × 10^5^/plate into 60 mm culture cell plates and allowed to grow for 24 h. Then, the cells were incubated for a further 40 h in the presence of different concentrations of test complex and cisplatin. Following treatment, the cells were harvested and resuspended in JC-1 Working Solution, according to the manufacturer’s protocol. After incubation for 30 min at 37 °C in the dark, cells were washed in PBS, resuspended and immediately analyzed by a FACSCanto II flow cytometer (Becton-Dickinson, Mountain View, CA, USA).

### 3.11. Evaluation of Apoptotic Cell Death by Annexin V-FITC and Propidium Iodide Staining

To detect phosphatidylserine translocation from the inner to the outer surface of the plasma membrane in A2780cis cells, a FITC Annexin V Apoptosis Detection Kit I (BD Pharmigen) was used. Briefly, 3 × 10^5^ cells were seeded into 60 mm cell culture plates overnight in complete growth medium, then serial dilutions of the complex were added. Following 40 h of incubation in the presence of test complex, cells were trypsinized, and 5 × 10^5^ cells were resuspended in 0.500 mL of binding buffer (0.01 M HEPES/NaOH (pH 7.4), 0.14 M NaCl, 2.5 mM CaCl_2_), Annexin V-FITC and propidium iodide (PI), as indicated by the supplier’s instructions. After incubation in the dark at room temperature for 15 min, the cytofluorimetric analysis of each sample was promptly performed by the FACSCanto II instrument (Becton–Dickinson, Mountain View, CA, USA).

## 4. Conclusions

Two new *trans*-dichloro(*N*,*N*-dialkylamino)Pt(II) complexes (**1** and **2**) containing the tryphenylarsine ligand were synthesized and characterized. The comparative analysis of the biological properties with the *trans*-dichloro(*N*,*N*-dialkylamino)Pt(II) triphenylphosphine analogues (3 and 4, respectively) allowed to conclude that the insertion of the *N*-butyl,*N*-benzylamino side chains as well as the triphenylarsine scaffold (complex **1**) induced a remarkable increase of the antiproliferative effect on ovarian carcinoma cells resistant to cisplatin.

Indeed, complex **1** (*trans*-dichloro(*N*-butyl,*N*-benzylamino)(triphenilarsino)Pt(II)) appears to be the most interesting complex, showing lower GI_50_ values compared to the related complexes on both sensitive and resistant ovarian carcinoma cells. The investigation into the mechanism of action identified topoisomerase II and mitochondria as possible intracellular targets, and a remarkable uptake ability in resistant A2780cis cells was found.

The different targets with respect to cisplatin and the significant cell accumulation, likely mediated by a passive diffusion mechanism, favored by the lipophilicity of the complex, may contribute to the overall biological effect exerted on resistant ovarian carcinoma cells and makes complex **1** a scaffold worthy of further development, with the aim to design more effective Pt(II) complexes.

## Figures and Tables

**Table 1:** Selected bond lengths and angles for *cis*-[PtCl_2_(AsPh_3_)(NCMe)].

Bond Length (Å)		Bond Angle (°)	
Pt(1)-N(1)	1.969 (3)	N(1)-Pt(1)-Cl(2)	177.32 (11)
Pt(1)-Cl(2)	2.2624 (11)	N(1)-Pt(1)-Cl(1)	88.16 (11)
Pt(1)-Cl(1)	2.3462 (10)	Cl(2)-Pt(1)-Cl(1)	90.43 (4)
Pt(1)-As(1)	2.3610 (4)	N(1)-Pt(1)-As(1)	93.42 (10)
		Cl(2)-Pt(1)-As(1)	88.14 (3)
		Cl(1)-Pt(1)-As(1)	175.98 (3)

**Table 2:** Antiproliferative effect induced by **1** and **2** after 72 h of incubation. The triphenylphosphine analogues 3 and 4 were shown for comparison and cisplatin was tested as a reference drug.

GI_50_ (µM) ^1^						
Complex	A2780	A2780cis	HeLa	HT-29	A549	Met-5A
**1**	0.49 ± 0.18	1.85 ± 0.16	5.75 ± 0.80	4.42 ± 0.78	4.48 ± 0.48	1.66 ± 0.84
**2**	3.66 ± 1.36	6.62 ± 1.50	10.9 ± 1.9	8.70 ± 1.80	17.9 ± 2.0	4.24 ± 0.75
3 ^2^	3.55 ± 0.26	3.63 ± 0.21	5.31 ± 0.65	nd	nd	
4 ^3^	nd	nd	0.42 ± 0.06	nd	2.3 ± 0.7	
cisplatin	1.08 ± 0.14	6.64 ± 0.77	1.42 ± 0.20	3.07 ± 1.05	2.45 ± 0.25	

^1^ Concentration of complex inducing a 50% reduction in cell growth. ^2^ Taken from Ref. [18]. ^3^ Taken from Ref. [22]. nd = not determined.

**Table 3:** Crystal data for *cis*-[PtCl_2_(AsPh_3_)(NCMe)].

Identification code	Shelx	
Empirical formula	C_20_H_18_AsCl_2_NPt	
Formula weight	613.26	
Temperature	296(2) K	
Wavelength	0.71073 Å	
Crystal system	Triclinic	
Space group	P-1	
Unit cell dimensions	a = 9.1126(5) Å	α = 62.7160(10)°.
	b = 10.9018(6) Å	β = 77.1370(10)°.
	c = 11.6047(7) Å	γ = 84.6470(10)°.
Volume	998.83(10) Å^3^	
Z	2	
Density (calculated)	2.039 Mg/m^3^	
Absorption coefficient	8.93 mm^−1^	
F(000)	580	
Crystal size	0.342 × 0.259 × 0.212 mm^3^	
Theta range for data collection	3.348 to 30.931°.	
Index ranges	−13 <= h <= 13, −15 <= k <= 15, −16 <= l <= 16	
Reflections collected	35,972	
Independent reflections	6010 [R(int) = 0.0322]	
Completeness to theta = 25.242°	99.8%	
Absorption correction	Numerical	
Max. and min. transmission	0.2768 and 0.1532	
Refinement method	Full-matrix least-squares on F^2^	
Data/restraints/parameters	6010/0/227	
Goodness-of-fit on F^2^	1.046	
Final R indices [I > 2sigma(I)]	R1 = 0.0327, wR2 = 0.0853	
R indices (all data)	R1 = 0.0363, wR2 = 0.0884	
Extinction coefficient	n/a	
Largest diff. peak and hole	2.063 and −1.809 Å^−3^	

## Data Availability

The data presented in this study are available in article and Supplementary Materials.

## Supplementary Materials

The following are available online, Figure S1: ^1^H NMR spectrum of *cis,trans*-[PtCl_2_(AsPh_3_)(NCMe)]. Figure S2: ^195^Pt NMR spectrum of *cis,trans*-[PtCl_2_(AsPh_3_)(NCMe)]. Figure S3: ^1^H NMR spectrum of **1**. Figure S4: ^13^C NMR spectrum of **1**. Figure S5: ^195^Pt NMR spectrum of **1**. Figure S6: ^1^H NMR spectrum of **2**. Figure S7: ^13^C NMR spectrum of **2**. Figure S8: ^195^Pt NMR spectrum of **2**. Figure S9: ^1^H NMR spectrum of **1** in DMSO-d6 solution after 24 h. Aromatic portion is omitted. Figure S10: Dot plots from a representative experiment of A2780cis cells incubated with complex **1** and loaded with JC-1. Figure S11: Dot plots from a representative experiment of A2780 cells incubated with complex **1** and loaded with JC-1. Figure S12: Dot plots from a representative experiment of A2780cis cells incubated with complex **1** and loaded with Annexin V-FITC and propidium iodide. Figure S13: Dot plots from a representative experiment of A2780 cells incubated with complex **1** and loaded with Annexin V-FITC and propidium iodide. CCDC 2109527 for *cis*-[PtCl_2_(AsPh_3_)(NCMe)] contains the supplementary crystallographic data for this paper. These data can be obtained free of charge from The Cambridge Crystallographic Data Centre.
