# Supplementary material for: trans-Dichloro(triphenylarsino)(N,N-dialkylamino)platinum(II) Complexes: In Search of New Scaffolds to Circumvent Cisplatin Resistance
Source: Molecules. 2022 Jan 19;27(3):644. doi: 10.3390/molecules27030644 (PMC8838190; doi:10.3390/molecules27030644)
Supplement: Supplementary file 1 [file molecules-27-00644-s001.zip › molecules-1531228-supplementary.pdf]

Supplementary Materials

trans-Dichloro(triphenylarsino)(*N,N*-dialkylamino)platinum(II) Complexes: In Search of New Scaffolds to Circumvent Cisplatin Resistance

Mariafrancesca Hyeraci [1,†], Laura Agnarelli [2,†], Luca Labella [2,3], Fabio Marchetti [2], Maria Luisa Di Paolo [4], Simona Samaritani [2,3] and Lisa Dalla Via [1,*]

[1] Department of Pharmaceutical and Pharmacological Sciences, Università degli Studi di Padova, Via F. Marzolo, 5, 35131 Padova, Italy; mariafrancesca.hyeraci@studenti.unipd.it (M.H.); lisa.dallavia@unipd.it (L.D.V.)
[2] Department of Chemistry and Industrial Chemistry, University of Pisa, Via G. Moruzzi 13, 56124 Pisa, Italy; laura.agnarelli@cpfs.mpg.de (L.A.); luca.labella@unipi.it (L.L.); fabio.marchetti1950@unipi.it (F.M.); simona.samaritani@unipi.it (S.S.)
[3] CISUP-Center for the Integration of Scientific Instruments, University of Pisa, 56126 Pisa, Italy
[4] Department of Molecular Medicine, Università degli Studi di Padova, Via G. Colombo, 3, 35131 Padova, Italy; marialuisa.dipaolo@unipd.it
* Correspondence: lisa.dallavia@unipd.it; Tel.: +39-049-8275712
† These authors contributed equally to this work.

Figure S1. ^{1}H NMR spectrum of *cis,trans*-$[PtCl_2(AsPh_3)(NCMe)]$.

Figure S2. ^{195}Pt NMR spectrum of *cis,trans*-$[PtCl_2(AsPh_3)(NCMe)]$.

Figure S3. ^{1}H NMR spectrum of **1**.

Figure S4. ^{13}C NMR spectrum of **1**.

Figure S5. ^{195}Pt NMR spectrum of **1**.

Figure S6. ^{1}H NMR spectrum of **2**.

Figure S7. ^{13}C NMR spectrum of **2**.

Figure S8. ^{195}Pt NMR spectrum of **2**.

Figure S9. ^{1}H NMR spectrum of **1** in DMSO-d6 solution after 24 h. Aromatic portion is omitted.

Figure S10. Dot plots from a representative experiment of A2780cis cells incubated with complex **1** and loaded with JC-1. A2780cis cells were incubated for 40 h with complex **1** or cisplatin (cisPt) at indicated concentrations. Percentages of cells with high (Q2) and low (Q4) mitochondrial membrane potential, are reported.

Figure S11. Dot plots from a representative experiment of A2780 cells incubated with complex **1** and loaded with JC-1. A2780 cells were incubated for 40 h with complex **1** at 5 M concentration. Percentages of cells with high (Q2) and low (Q4) mitochondrial membrane potential, are reported.

Figure S12. Dot plots from a representative experiment of A2780cis cells incubated with complex **1** and loaded with Annexin V-FITC and propidium iodide. A2780cis cells were incubated for 40 h with complex **1** at indicated concentrations. Percentages of viable (Q3), apoptotic (Q2+Q4) and necrotic (Q1) A2780cis are reported.

Figure S13. Dot plots from a representative experiment of A2780 cells incubated with complex **1** and loaded with Annexin V-FITC and propidium iodide. A2780 cells were incubated for 40 h with complex **1** at 10 M concentration. Percentages of viable (Q3), apoptotic (Q2+Q4) and necrotic (Q1) A2780cis are reported.

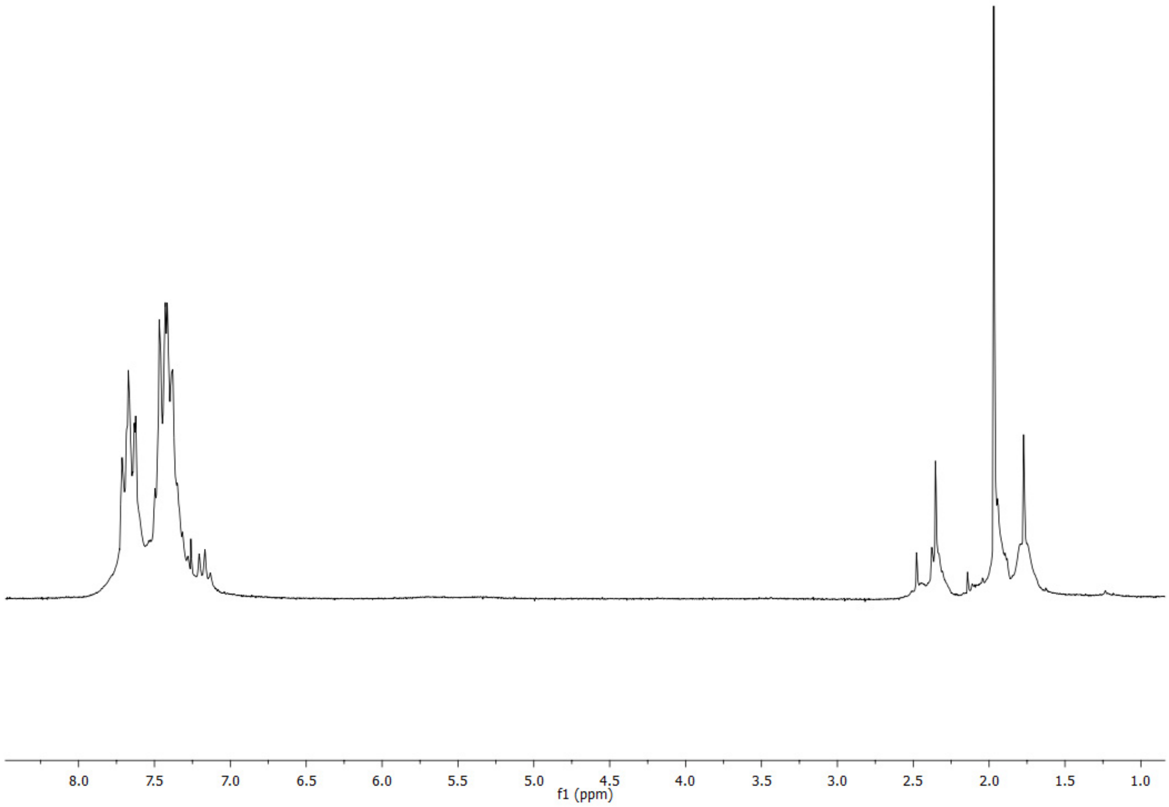

Figure S1. ^{1}H NMR spectrum of *cis,trans*-[$PtCl_2(AsPh_3)(NCMe)$].

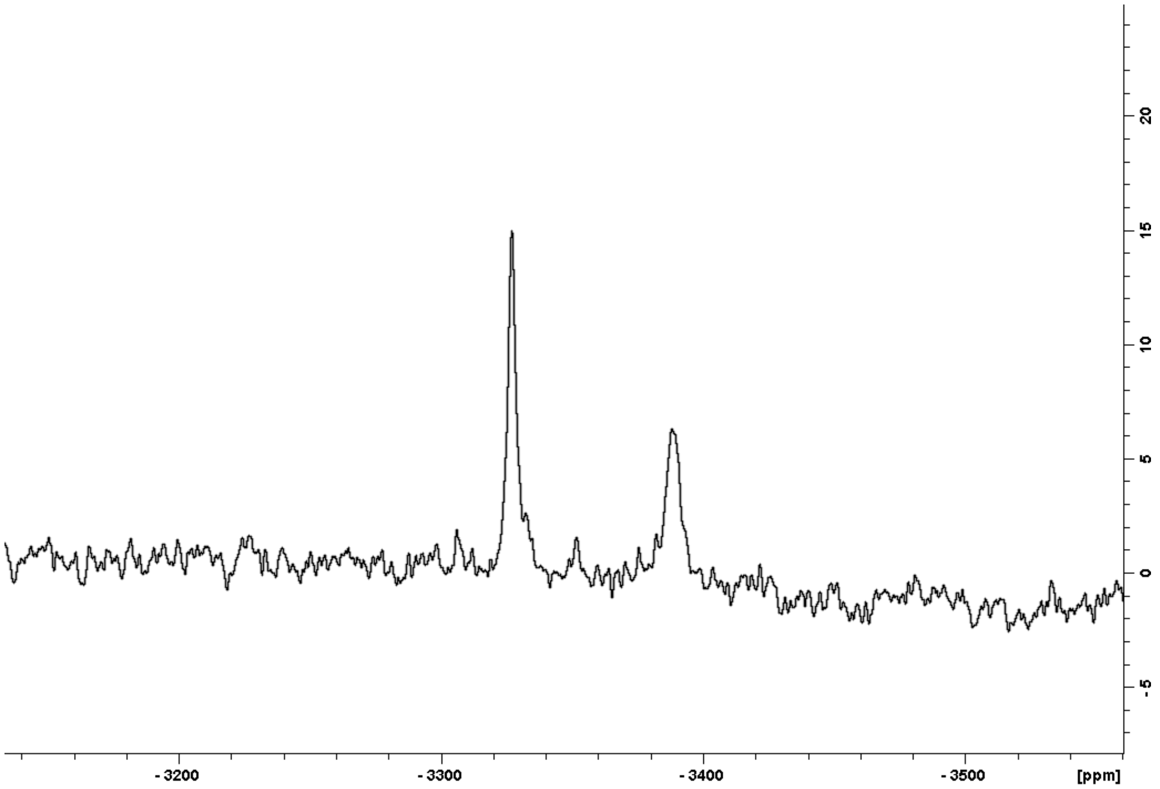

Figure S2. ^{195}Pt NMR spectrum of *cis,trans*-[$PtCl_2(AsPh_3)(NCMe)$].

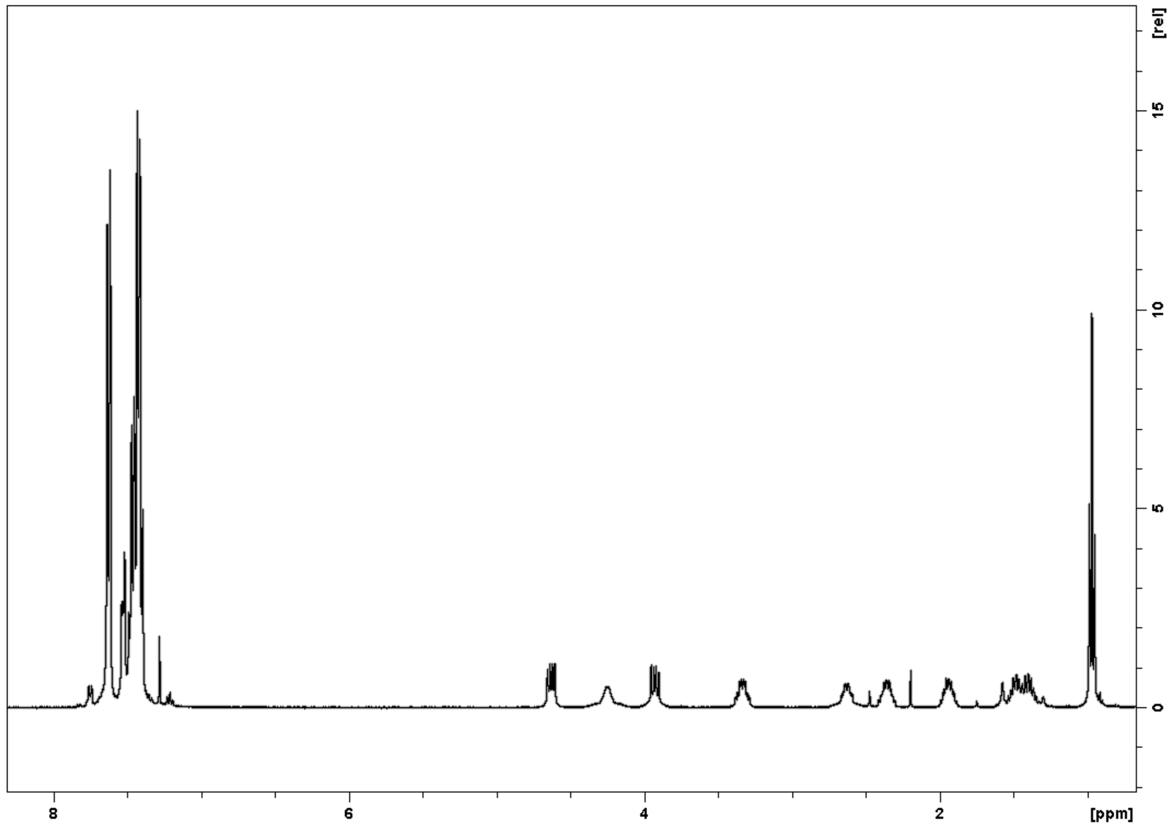

Figure S3. ^{1}H NMR spectrum of **1**.

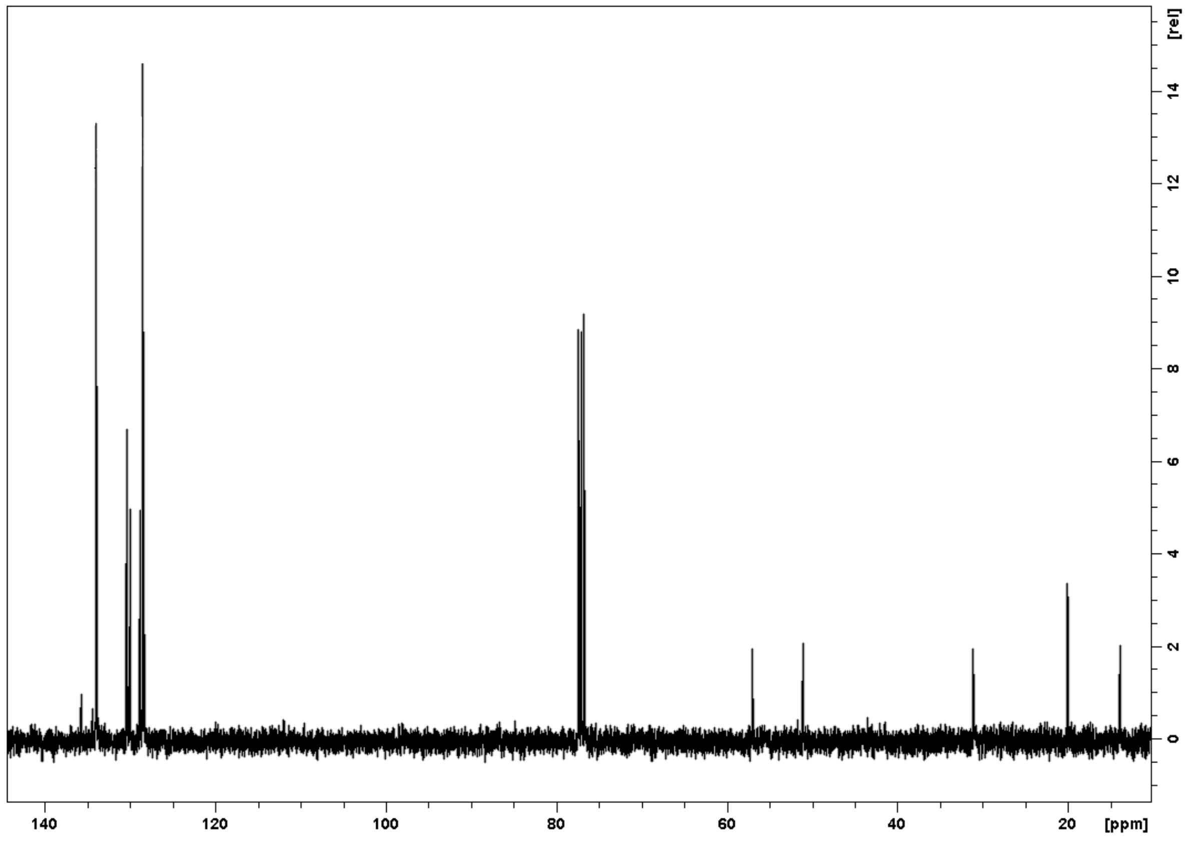

Figure S4. ^{13}C NMR spectrum of **1**.

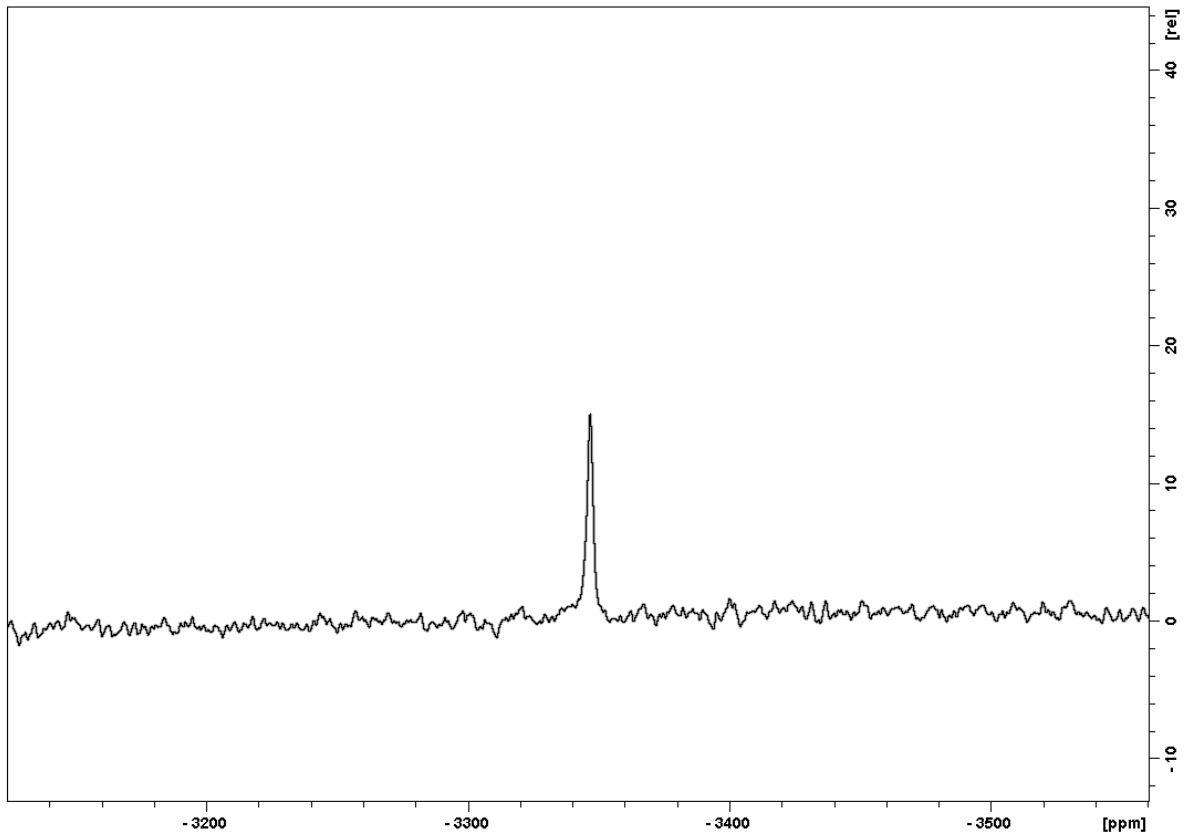

Figure S5. ^{195}Pt NMR spectrum of **1**.

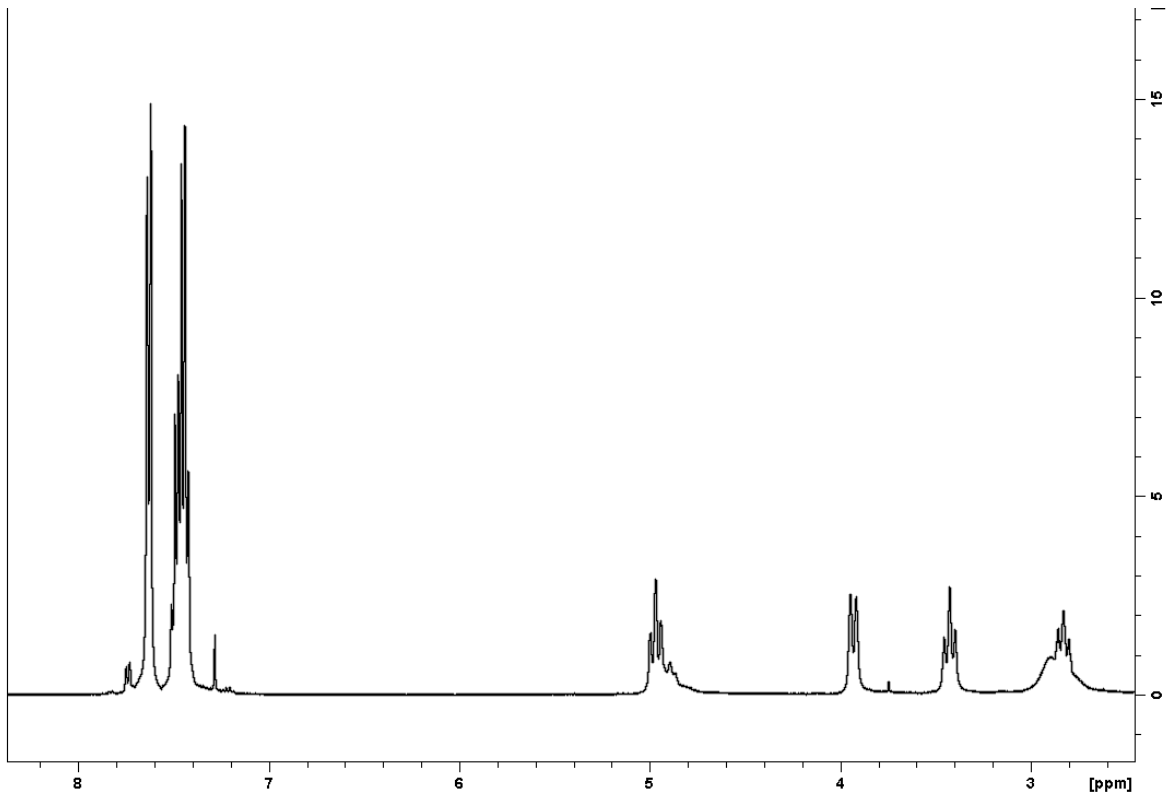

Figure S6. ^{1}H NMR spectrum of **2**.

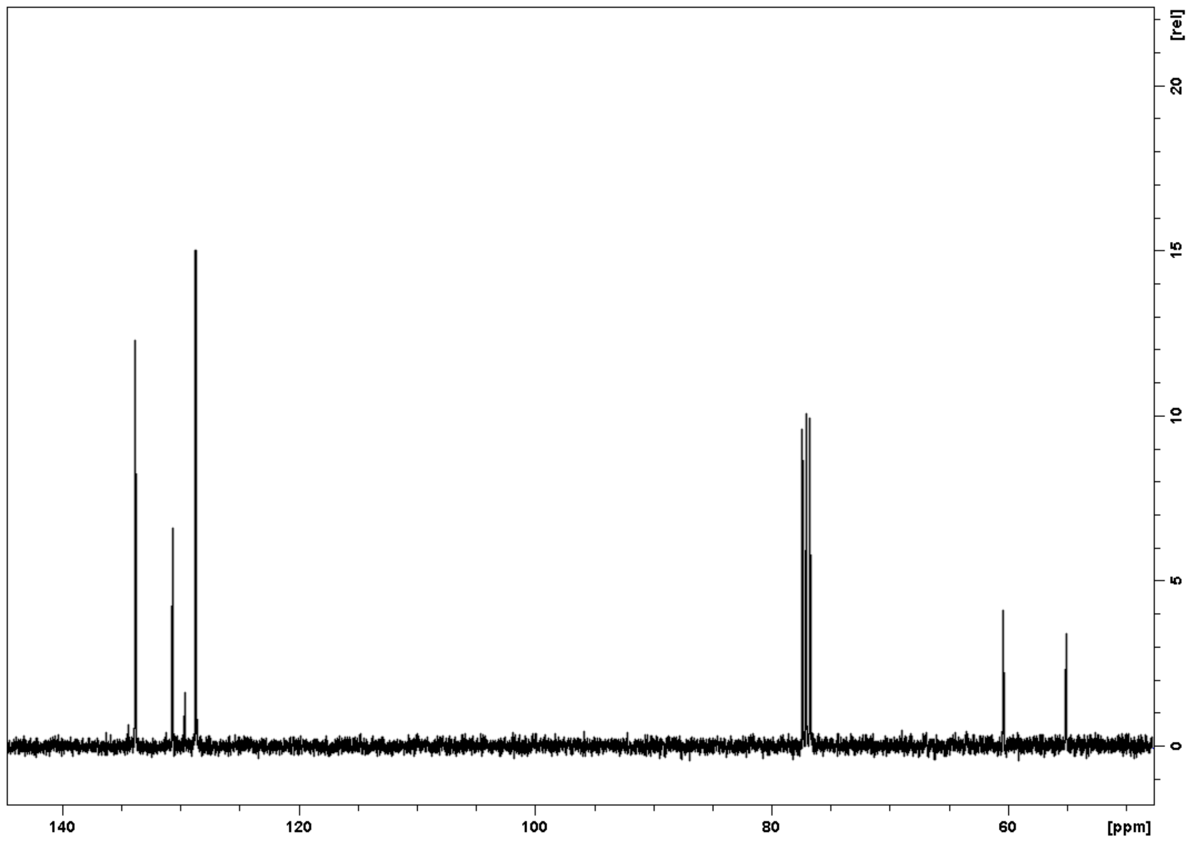

Figure S7. ^{13}C NMR spectrum of **2**.

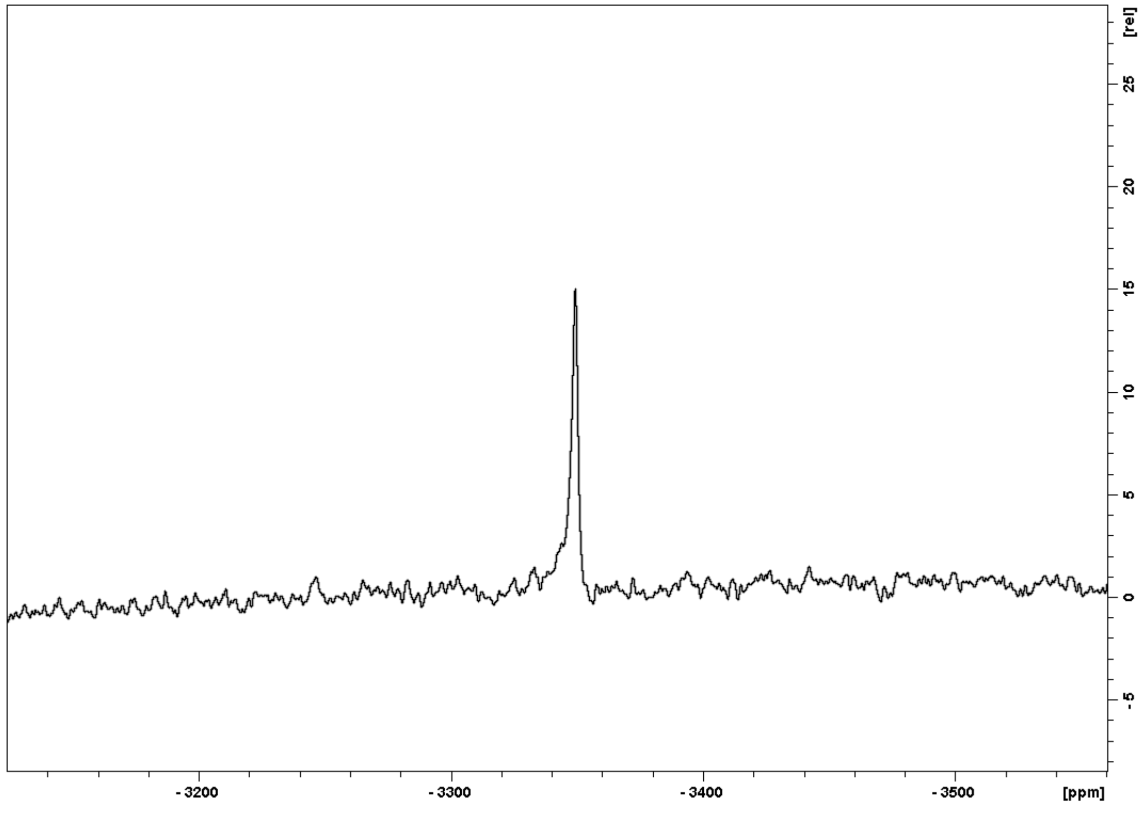

Figure S8. ^{195}Pt NMR spectrum of **2**.

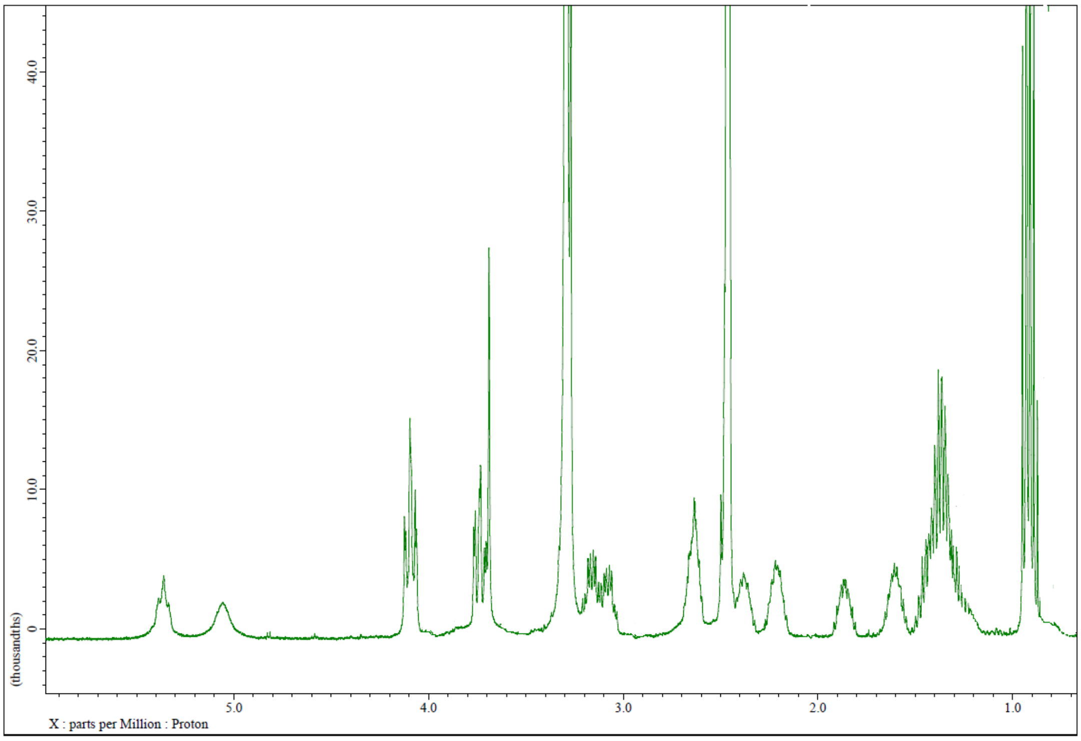

Figure S9. ^{1}H NMR spectrum of **1** in DMSO-d6 solution after 24 h. Aromatic portion is omitted.

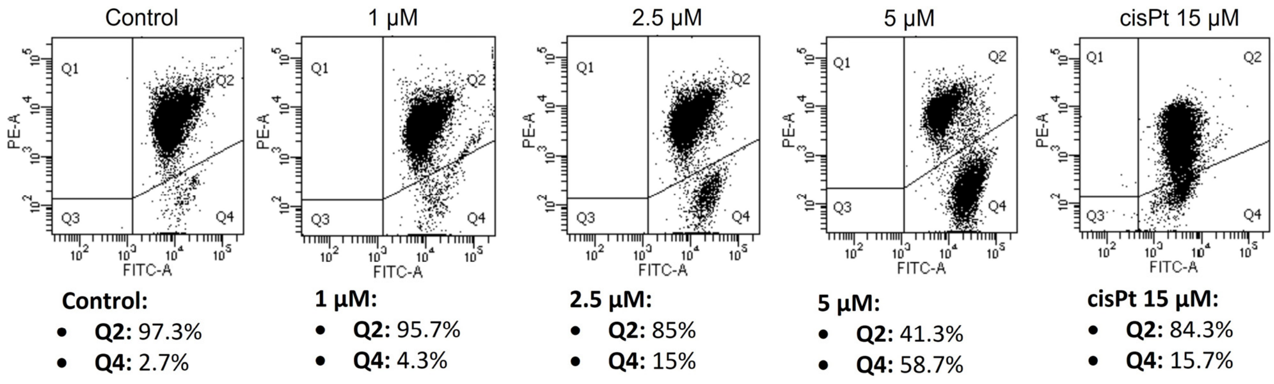

Figure S10. Dot plots from a representative experiment of A2780cis cells incubated with complex **1** and loaded with JC-1. A2780cis cells were incubated for 40 h with complex **1** or cisplatin (cisPt) at indicated concentrations. Percentages of cells with high (Q2) and low (Q4) mitochondrial membrane potential, are reported.

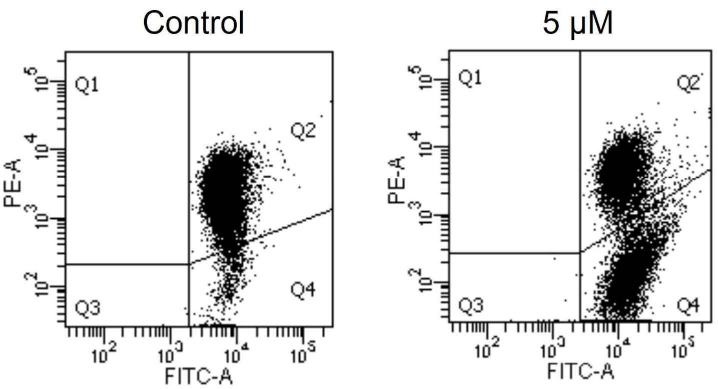

Control:
- **Q2:** 94.4%
- **Q4:** 5.6%

5 μM:
- **Q2:** 46.1%
- **Q4:** 53.9%

Figure S11. Dot plots from a representative experiment of A2780 cells incubated with complex **1** and loaded with JC-1. A2780 cells were incubated for 40 h with complex **1** at 5 M concentration. Percentages of cells with high (Q2) and low (Q4) mitochondrial membrane potential, are reported.

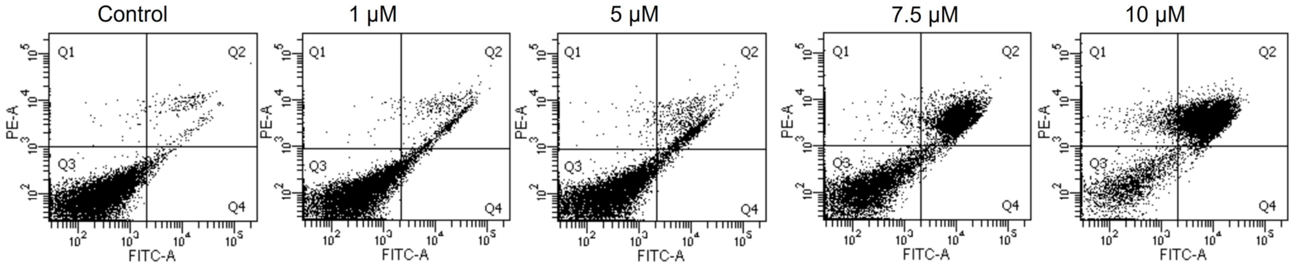

Control:
- **Q1:** 0.6%
- **Q2+Q4:** 6.4%
- **Q3:** 93%

1 μM:
- **Q1:** 0.6%
- **Q2+Q4:** 11.4%
- **Q3:** 88%

5 μM:
- **Q1:** 1.5%
- **Q2+Q4:** 12.3%
- **Q3:** 86.2%

7,5 μM:
- **Q1:** 3.7%
- **Q2+Q4:** 35.5%
- **Q3:** 60.8%

10 μM:
- **Q1:** 19%
- **Q2+Q4:** 57%
- **Q3:** 24%

Figure S12. Dot plots from a representative experiment of A2780cis cells incubated with complex **1** and loaded with Annexin V-FITC and propidium iodide. A2780cis cells were incubated for 40 h with complex **1** at indicated concentrations. Percentages of viable (Q3), apoptotic (Q2+Q4) and necrotic (Q1) A2780cis are reported.

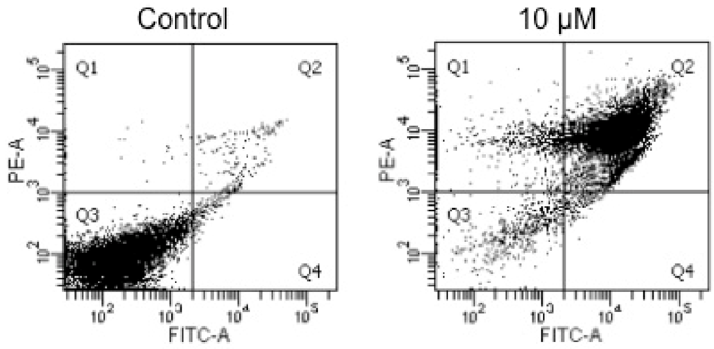

Control:
- **Q1:** 0.4%
- **Q2+Q4:** 3%
- **Q3:** 96.6%

10 µM:
- **Q1:** 15%
- **Q2+Q4:** 80.8%
- **Q3:** 4.2%

Figure S13. Dot plots from a representative experiment of A2780 cells incubated with complex **1** and loaded with Annexin V-FITC and propidium iodide. A2780 cells were incubated for 40 h with complex **1** at 10 M concentration. Percentages of viable (Q3), apoptotic (Q2+Q4) and necrotic (Q1) A2780cis are reported.
